# Neurophysiological and neuroradiological test for early poor outcome (Cerebral Performance Categories 3–5) prediction after cardiac arrest: Prospective multicentre prognostication data

**DOI:** 10.1016/j.dib.2019.104755

**Published:** 2019-11-04

**Authors:** Maenia Scarpino, Francesco Lolli, Giovanni Lanzo, Riccardo Carrai, Maddalena Spalletti, Franco Valzania, Maria Lombardi, Daniela Audenino, Maria Grazia Celani, Alfonso Marrelli, Sara Contardi, Adriano Peris, Aldo Amantini, Claudio Sandroni, Antonello Grippo, for the ProNeCAStudy Group

**Affiliations:** a SODc Neurofisiopatologia, Dipartimento Neuromuscolo-Scheletrico e degli Organi di Senso, AOU Careggi, Florence, Italy; b IRCCS, Fondazione Don Carlo Gnocchi, Florence, Italy; c Dipartimento di Scienze Biomediche Sperimentali e Cliniche, Università degli Studi di Firenze, Italy; d UO Neurofisiopatologia, Arcispedale Santa Maria Nuova, Reggio nell’Emilia, Italy; e UO Neurologia, Ospedale San Giuseppe, Empoli, Italy; f SC Neurologia, EO Ospedale Galliera, Genova, Italy; g UO Neurofisiopatologia, Ospedale Santa Maria della Misericordia, Perugia, Italy; h UOC Neurofisiopatologia, Ospedale San Salvatore, L'Aquila, Italy; i NeurofisiopatologiaInterventiva, Ospedale civile di Baggiovara, Modena, Italy; j Unità di Terapia Intensiva, Dipartimento Neuromuscolo-Scheletrico e degli Organi di Senso, AOU Careggi, Florence, Italy; k Istituto Anestesiologia e Rianimazione Università Cattolica del Sacro Cuore, Fondazione Policlinico Universitario “Agostino Gemelli” IRCCS, Rome, Italy

**Keywords:** Cardiac arrest, Anoxia-ischemia, Brain, Coma, Prognosis, Electroencephalogram, Somatosensory evoked potentials, Computed tomography

## Abstract

The data presented here are related to our research article entitled “Neurophysiology and neuroimaging accurately predict poor neurological outcome within 24 hours after cardiac arrest: a prospective multicentre prognostication study (ProNeCA)” [1].

We report a secondary analysis on the ability of somatosensory evoked potentials (SEPs), brain computed tomography (CT) and electroencephalography (EEG) to predict poor neurological outcome at 6 months in 346 patients who were comatose after cardiac arrest. Differently from the related research article, here we included cerebral performance category (CPC) 3 among poor outcomes, so that the outcomes are dichotomised as CPC 1–2 (absent to mild neurological disability: good outcome) vs. CPC 3–5 (severe neurological disability, persistent vegetative state, or death: poor outcome). The accuracy of the index tests was recalculated accordingly. A bilaterally absent/absent-pathological amplitude (AA/AP) N20 SEPs wave, a Grey Matter/White Matter (GM/WM) ratio <1.21 on brain CT and an isoelectric or burst suppression EEG predicted poor outcome with 49.6%, 42.2% and 29.8% sensitivity, respectively, and 100% specificity. The distribution of positive results of the three predictors did not overlap completely in the population of patients with poor outcome, so that when combining them the overall sensitivity raised to 61.2%.

**Table:** Specifications Table

Subject area	*Cardiac Arrest(CA)*
More specific subject area	*Multimodal Neurological Prognostication in comatose patients after cardiac arrest (CA).*
Type of data	*Quantitative data of index tests, figures and tables*
How data was acquired	*Be-Plus Galileo for acquisition of EEG and SEP.*
Data format	*Raw data, continuous and categorical variables*
Experimental factors	*Electroencephalography (EEG) patterns classified according to the American Clinical Neurophysiology Society (ACNS) terminology; Somatosensory Evoked Potentials (SEPs) classified according to the cortical responses on both hemispheres; Grey Matter/White Matter (GM/WM) ratio density on Brain Computed Tomography (CT).*
Experimental features	*Tests were performed within the first 24 hours after CA. The primary endpoint was neurological outcome at* 6 months*, measured using the Cerebral Performance Categories (CPC). Accuracy was measured using sensitivity, specificity and Receiving Operating Characteristic (ROC) curve where appropriate.*
Data source location	*Italy (Florence, Modena, Empoli, Reggio Emilia, L'Aquila, Perugia, Genoa).*
Data accessibility	*Summary data are available in this article. Excel data file is attached as*supplementary material
Related research article	*Neurophysiology and neuroimaging accurately predict poor neurological outcome within 24 hours after cardiac arrest: a prospective multicentre prognostication study (ProNeCA). Scarpino* et al.*, Resuscitation. 2019,* 143:115–123.

**Table:** 

**Value of the Data** • A description of which SEP, EEG and brain CT features are relevant for neurological prognostication in CA comatose surviving patients and a description of the statistical analysis. • A statistical analysis including patients with severe disability (CPC 3) in the poor outcome group (a neurological outcome aggregation similar to most of previous published data). This aggregation allows a generalizability of the data. • These data has been derived from a population of patients where withdrawal of life sustaining treatment (WLST) based on prognostication of poor neurological outcome was not performed, except when brain death occurred. • In most of previous prognostication data censoring from WLST, which is often based on the same predictors under investigation, has an important confounding effect.

## 1 Data

**Fig. 1:**
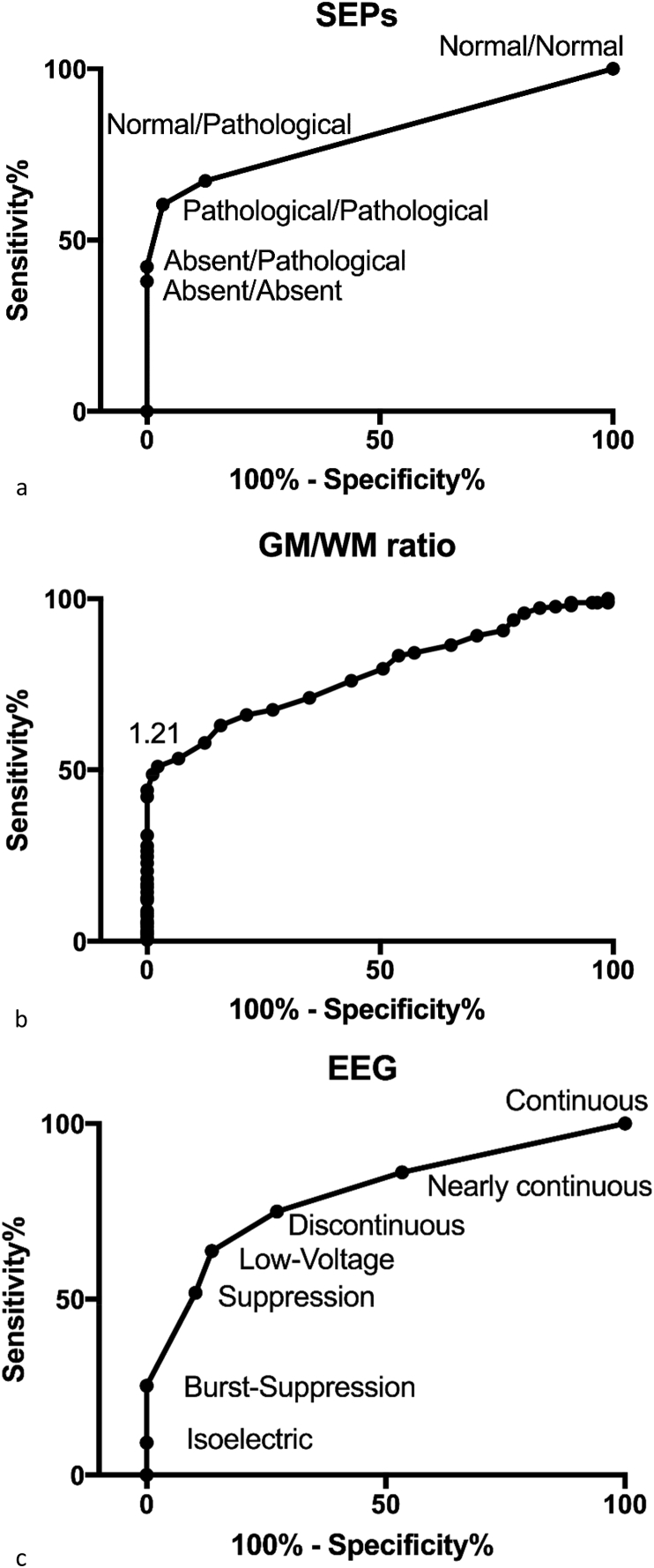
a-c. ROC curves showing the accuracy in prediction of poor prognosis for SEP patterns (a) GM/WM ratio on brain CT (b) and EEG patterns (c). Cerebral outcome categories 3, 4 and 5 correspond to poor outcome. The x axis shows the sensitivity of the tests, ranging from 0 to 1.0 (0–100%), while the y axis shows the percentage of false positive results (100% - specificity).

**Fig. 2:**
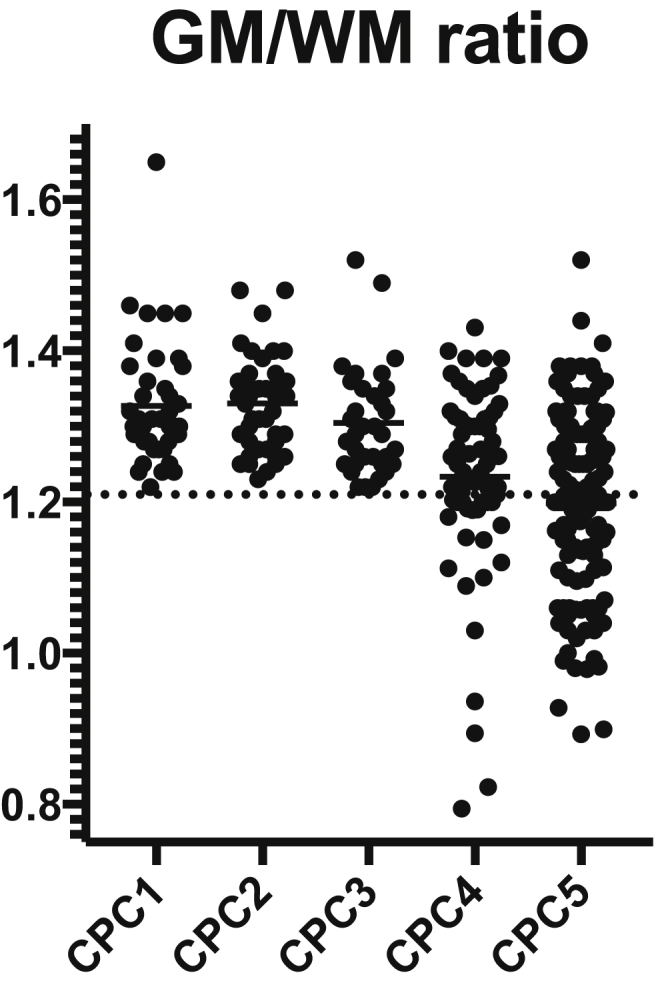
Scatterplot showing the distribution of GM/WM ratio according to the Cerebral Outcome Categories (CPC). Closed circles correspond to individual patient data.

Table 1 shows the demographic characteristics of the 346 enrolled patients. Based on ROC curve analysis (Fig. 1a–c), the optimal cut-off for SEPs, EEG, and brain CT that maximised sensitivity for poor outcome prediction while maintaining 100% specificity was identified, and index test data were dichotomised accordingly. SEP patterns were dichotomised as grade 2 (AA-AP) vs. grade 1 (NN-NP-PP). EEG patterns were dichotomised as malignant (isoelectric, burst-suppression) vs. non-malignant (continuous, nearly continuous, discontinuous, epileptic discharges, low-voltage, and suppression), whereas for brain CT a threshold of <1.21 of GM/WM ratio was identified (Fig. 2). Grade 2 SEP predicted poor outcome with 49.6% sensitivity and 100% (CI 43.3–55.8) specificity (AUC = 0.86; CI 0.82–0.89). A GM/WM ratio <1.21 predicted poor outcome with 42.2% sensitivity and 100% (CI 36.1–48.5) specificity (AUC = 0.78; CI 0.73–0.83). The cut-off ensuring 100% specificity for EEG was identified as malignant pattern (isoelectric, burst suppression) vs. non-malignant patterns (continuous, nearly continuous, discontinuous, epileptic discharges, low-voltage, suppression). Malignant patterns predicted poor outcome with 29.8% (CI 24.3–35.8) sensitivity and 100% specificity (AUC = 0.90; CI 0.86–0.93). Data are reported in Table 2. As a further analysis we combined the data of the three tests and calculated the cumulative proportion of patients who were correctly identified as having poor outcome (CPC 3–5) when at least one among the three investigated indices reached the threshold for 100% specificity. According to our data, 166 patients had at least one poor prognostic criterion.

**Table 1:** Characteristics of the study population.

	Patient Included n = 346
**Age, years**	68 (48–70)
**Gender, female**	130 (37)
**Out-of-hospital**	273 (78)
**Witnessed**	282 (82)
**CA duration (min)**	15 (9–28)
**Initial rhythm**	
VF/pVT	144 (42)
PEA	87 (25)
Asystole	71 (21)
Unknown	44 (12)
**Pupillary reflex at neurophysiological evaluation**	
Absent	147 (42)
Present	189 (55)
Unknown	10 (3)
**GCS at ICU admission**	3 (3–8)
**TTM**	
No	207 (60)
34 °C	123 (35)
36 °C	16 (5)
**CPC at hospital discharge**	
CPC 1	11 (3)
CPC 2	23 (7)
CPC 3	62 (18)
CPC 4	136 (39)
CPC 5	114 (33)
**CPC at 6 months**	
CPC 1	43 (12)
CPC 2	45 (13)
CPC 3	35 (10)
CPC 4	68 (20)
CPC 5	155 (45)

Data are presented as count (percentage) or median (interquartile range; range for GCS score). CPC, Cerebral Performance Categories; GCS, Glasgow Coma Scale; ICU, intensive care unit; PEA, pulseless electrical activity; pVT, pulseless ventricular tachycardia; VF, ventricular Fibrillation; TTM, Targeted Temperature Management.

**Table 2:** Accuracy of index tests (single and in combination) for prediction of poor (CPC 3-4-5) outcome at 6 months.

Index test	TP	FP	TN	FN	Sensitivity % (95%CI)	False positive rate % (95%CI)
**Single test prediction**						
Grade 2 SEPs	128	0	88	130	49.6 (43.3–55.8)	0 (0–4)
GM/WM ratio < 1.21 on brain CT	109	0	88	149	42.2 (36.1–48.5)	0 (0–4)
Malignant EEG	77	0	88	181	29.8 (24.3–35.8)	0 (0–4)
**Combination of two tests**						
Grade 2 SEPs or GW/WM ratio < 1.21	157	0	88	101	60.8 (54.6–66.8)	0 (0–4)
Malignant EEG or GW/WM ratio < 1.21	116	0	88	142	44.9 (38.7–51.2)	0 (0–4)
Grade 2 SEPs or Malignant EEG	129	0	88	129	50.0 (43.7–56.2)	0 (0–4)
**Combination of three tests**						
At least one test predicting poor outcome	158	0	88	100	61.2 (55.0–67.2)	0 (0–4)

CI: Confidence Interval; CT: computed tomography; EEG: Electroencephalogram; GW/WM: Gray Matter/White Matter; SEPs: Somatosensory Evoked Potentials.

FN, false negative; FP, false positive; TN, true negative; TP, true positive.

**Fig. 3:**
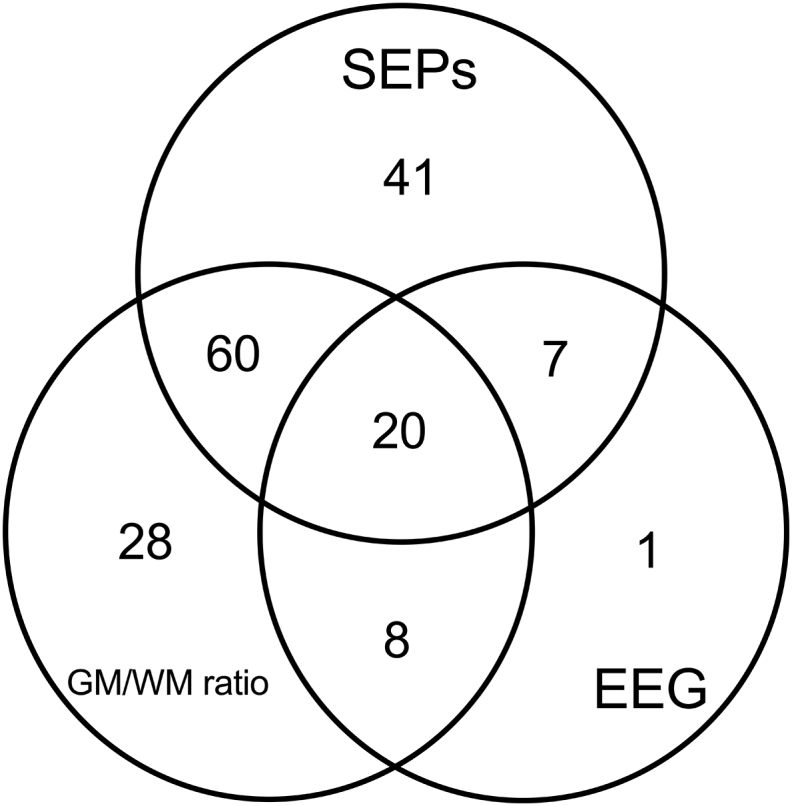
Venn diagram showing the distribution of index test results among true positives. Only 21 patients were detected by all three tests. The true positives identified by only one test were 41/128, 28/116, and 1/36 for SEPs, CT, and EEG, respectively.

The distribution of positive data of different tests overlapped only partially in patients with poor outcome. Only 21 of these patients were detected by all three tests. In 41/128 patients with positive SEP data, in 28/116 patents with positive brain CT data, and in 1/36 patients with positive EEG data true positives were identified by only one test (Venn diagram in Fig. 3). Consequently, the cumulative sensitivity increased by adding the individual test sensitivities. When two tests were considered, if at least one of the patterns predicting poor outcome was present, the sensitivity increased from 49.6% (obtained with the best single performing test, SEPs), to 60.8% (obtained by the combination of SEPs and brain CT). When all three tests were considered, the sensitivity for poor prognosis increased to 61.2%, while maintaining 100% specificity.

## 2 Experimental design, materials, and methods

### 2.1 Patient management

In all comatose patients included in the analysis, brain CT, EEG and SEPs were performed within 24 hours after CA. The data of these instrumental tests did not affect ongoing patient management. Patients were sedated using either propofol (range 1–2mg/Kg/h) or midazolam (range 0.03–0.1mg/Kg/h) as recommended. The choice of the targeted temperature management i.e., 34 °C vs. 36 °C was at the discretion of the participating centre [2].

### 2.2 Index tests

SEP analysis was based on the evaluation of the presence, amplitude [3]or absence of the cortical responses (N20/P25 complex) on both hemispheres [4]. We identified six SEP patterns: NN, NP, PP, AN, AP and AA, in which N stands for normal (N20/P25 amplitude is normal), P stands for pathological (N20/P25 amplitude is < 1.2μV or the difference between the two sides is greater than 50%) and A stands for absent if no reproducible cortical components could be identified in the presence of a lemniscal potential [5,6]. SEPs were elicited via stimulation of the median nerve at the wrist to an intensity of 4–5mA greater than that needed to evoke a muscular response. In the case of the use of neuromuscular blocking, Erb potential amplitude was used to estimate the intensity of the stimulation. Pulse duration was 0.2 m s and stimulus rate was 3 Hz. A portable digital 4-channel EPA apparatus was used. Recording stainless steel needle electrodes were placed at Erb's point (referred to contralateral Erb's point), spinous process CV7 (referred to the anterior neck) and C3 and C4 (referred to Fz and ipsilateral mastoid). At least two repetitions (averages of 300 responses) were needed to assess the reproducibility of waveforms. The analysis time was 100 m s and bandwidth was 5Hz–3 kHz. EEGs were classified according to the ACNS terminology for EEGs in critical care [7]. The “continuity” and the “voltage” of the background activity were the main parameters taken in to account for EEG classification. Thus, the main patterns identified were: continuous; nearly continuous; discontinuous; burst-suppression; suppression; epileptiform discharges, low voltage (voltage <20μV) and isoelectric. Isoelectric (voltage <2μV) recordings were identified, although the original classification did not distinguish them from suppressed activity (voltage <10μV) [8]. Brain CT prognostic power is based on the GM/WM ratio density. In particular in our analysis we performed density measurements limited to the basal ganglia level, according to previously reported method [[9], [10], [11]], as GM/WM ratio =(Caudate Nucleus + Putamen)/(Corpus Callosum + Posterior limb of the Internal Capsule). For further details regarding patient management, SEP and EEG recording and Brain CT acquisition refer to the related research article [1].

### 2.3 Ethical approval

The protocol was approved by the Regional Ethics Committee of Tuscany (Ref OSS.15.009). Written informed consent was obtained from the patient's authorized representative prior to the subject enrolment.

### 2.4 Statistical analysis

Continuous variables were reported as median and inter-quartile range (IQR), whereas categorical variables were reported as numbers and percentages. Normality of baseline distribution was tested using the Shapiro-Wilk test. The Pearson's chi-square and the Mann–Whitney U tests were used for comparing categorical and continuous variables, respectively. For these data, sensitivity and specificity of SEPs, EEG, and brain CT were calculated. In addition, receiver operating characteristics (ROC) curve and the corresponding area under the curve (AUC) are reported. When predicting poor outcome, maximising specificity is essential in order to avoid a falsely pessimistic prediction leading to treatment limitations in patients with a chance of neurological recovery [12,13]. Thus, data of the investigated predictors were dichotomised based on the value or category that ensured 100% specificity, as identified on the ROC curve. Both the individual and the combined prognostic accuracy of index tests have been investigated. We performed a tree-based analysis to identify the best combination of different predictors in order to maximise sensitivity for outcome prediction, and calculated the sensitivity of the possible combinations of two criteria indicating poor outcome with 100% specificity. Neurological status was determined using CPC at two follow-up points: at hospital discharge, looking at the chart review, and, for patients surviving at hospital discharge, at least 6 months after CA, by telephone interview [14,15]. A *p*-value<0.05 was considered statistically significant. Statistical analysis was performed using Wizard 1.9 version (Evan Miller, USA) and IBM-SPSS Statistics for Windows 25.0 version (IBM Corp., Armonk, NY, USA).
